# Nanomedicine-induced programmed cell death in cancer therapy: mechanisms and perspectives

**DOI:** 10.1038/s41420-024-02121-0

**Published:** 2024-08-29

**Authors:** Lin Luobin, He Wanxin, Guo Yingxin, Zheng Qinzhou, Liang Zefeng, Wu Danyang, Li Huaqin

**Affiliations:** 1 School of Health Sciences, Guangzhou Xinhua University, 19 Huamei Road, Tianhe District Guangzhou, 510520, China; 2 School of Life Sciences and Biopharmaceuticals, Guangdong Pharmaceutical University, Guangzhou, 510006, China

**Keywords:** Drug development, Cancer, Cancer

## Abstract

The balance of programmed cell death (PCD) mechanisms, including apoptosis, autophagy, necroptosis and others, is pivotal in cancer progression and treatment. Dysregulation of these pathways results in uncontrolled cell growth and resistance to conventional therapies. Nanomedicine offers a promising solution in oncology through targeted drug delivery enabling precise targeting of cancer cells while preserving healthy tissues. This approach reduces the side effects of traditional chemotherapy and enhances treatment efficacy by engaging PCD pathways. We details each PCD pathway, their mechanisms, and innovative nanomedicine strategies to activate these pathways, thereby enhancing therapeutic specificity and minimizing harm to healthy tissues. The precision of nanotechnology in targeting PCD pathways promises significant improvements in cancer treatment outcomes. This synergy between nanotechnology and targeted PCD activation could lead to more effective and less toxic cancer therapies, heralding a new era in cancer treatment.

## Facts

- Active targeting enhances the specificity of the delivery system, ensuring direct delivery to the tumor cells and reducing off-target effects, thereby optimizing therapeutic outcomes.
- Nanoparticles can be engineered to directly induce apoptosis by delivering pro-apoptotic drugs or genes specifically to cancer cells, as well as other forms of programmed cell death (PCD).
- Nanoparticles can induce immunogenic cell death (ICD) by releasing of chemotherapeutic agents that trigger the emission of damage-associated molecular patterns (DAMPs) from dying cancer cells.

## Open Questions

- Given the biocompatibility concerns associated with nanomaterials, is it feasible to produce personalized nanomedicines using tissues obtained directly from patients?
- What approaches can be utilized to evaluate and ensure the long-term safety and effectiveness of nanomedicines in cancer therapy?
- Can ICD offer an immunological approach to cancer treatment by regulating PCD within various tumor microenvironments?
- Can nanomedicine be utilized to overcome multidrug resistance by targeting PCD pathways in cancer therapy?

## Introduction

Cancer remains one of the leading causes of mortality worldwide. Efforts aimed at developing treatments that are both more effective and less harmful have led to considerable interest in the application of nanotechnology in medicine. Traditional therapies have grappled with balancing efficacy with toxicity, frequently leading to substantial patient morbidity [1, 2]. Through the utilization of nanoparticles, nanomedicine significantly improves drug solubility, prolongs drug circulation, enhances tumor tissue penetration, and facilitates controlled drug release. These nanoparticles are meticulously engineered to target specific cancer cell markers, ensuring the precise delivery of therapeutic agents to tumor cells while mitigating harm to healthy tissues [3].

**Fig. 1:**
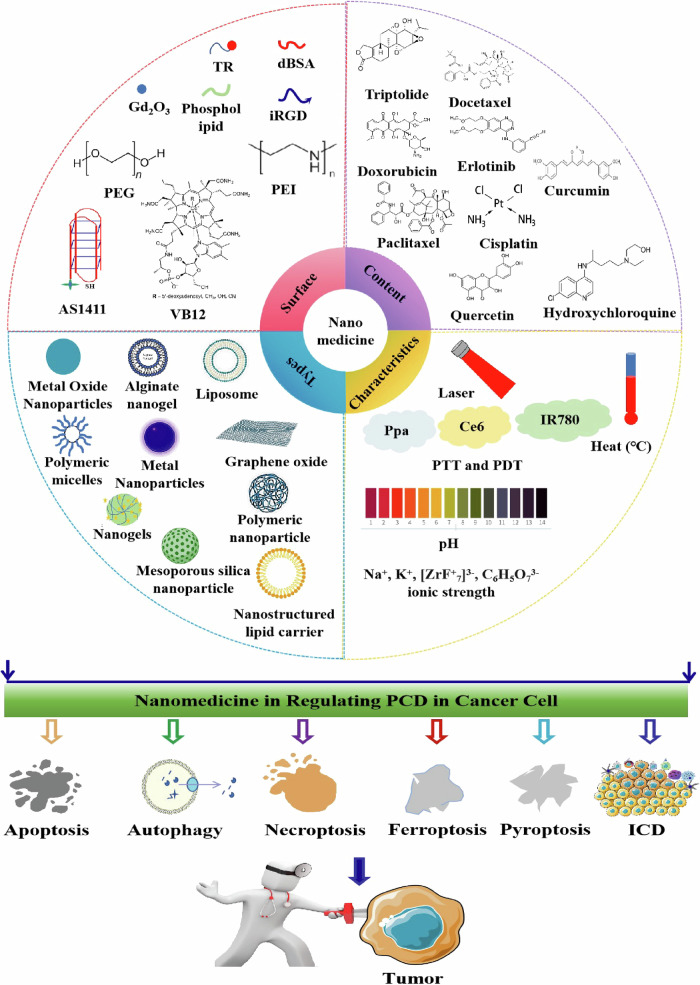
Components of nanomedicine strategies targeting the PCD for cancer therapy. Recent breakthroughs in nanotechnology offer innovative ways to modulate PCD mechanisms crucial in cancer progression and treatment, including apoptosis, autophagy, and necroptosis. Dysregulation of these pathways leads to uncontrolled cell proliferation and therapy resistance. Figure 1 depicts the application of nanomedicines targeting PCD pathways in cancer treatment. “Content” lists the drugs delivered by one or more types of nanocarriers. “Surface” represents ligands or functionalized structures on nanoparticles. “Types” indicates the types of nanomaterials. “Characteristics” describes characteristics of nanomedicines, such as pH-responsive release, PDT and PTT therapies, thermosensitive nanomedicines, and methods utilizing ion pressure for treatment. dBSA denatured Bovine Serum Albumin, TR tandem peptide TH-RGD, iRGD internalizing-RGD, PEG polyethylene glycol, PEI polyethyleneimine, VB12 vitamin B12, AS1411 nucleolin-targeted aptamers, Ppa pheophorbide A, Ce6 a near-infrared (NIR) photosensitizer, IR780 Indocyanine Green, ICD immunogenic cell death, PTT photothermal therapies, PDT photodynamic therapies.

The modulation of programmed cell death (PCD) pathways, such as apoptosis, autophagy, necroptosis and others, offers promising strategy for cancer treatment. Nanomedicine-induced immunogenic cell death (ICD) is also emerging as a promising strategy [4]. Cancer cells frequently develop resistance to avoid PCD, reducing treatment efficacy [5]. In this context, nanotechnology provides targeted delivery systems and mechanisms to directly modulate PCD pathways within the tumor microenvironment, enhancing therapy efficacy while minimizing side effects and overcoming drug resistance (Fig. 1).

This review investigates nanomedicine’s role in promoting PCD in cancer cells to enhance therapeutic outcomes while preserving normal tissues. By examining nanomedicine design, functionalization, and interactions of nanomedicine within cancer cells, as well as recent clinical trial findings, we will explore how nanomedicine triggers apoptosis, autophagy, necroptosis, and others PCD pathways in cancer cells. Additionally, we will also discuss the therapeutic potential of leveraging these pathways.

## Mechanistic pathways of nanomedicine in cancer therapy

**Fig. 2:**
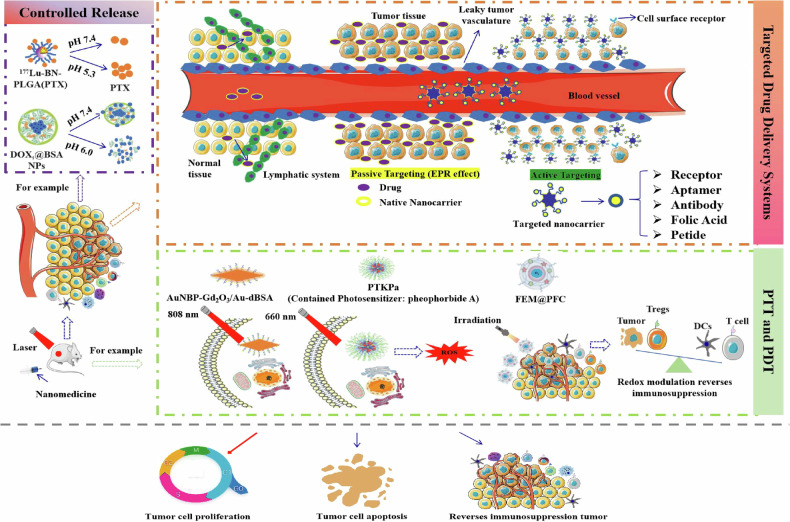
Application characteristics of nanomedicine in cancer therapy. This schematic illustrates the mechanisms of nanomedicine-based targeted drug delivery and tumor therapy. Nanomedicines utilize targeted delivery systems and controlled release technologies for precise drug delivery and real-time release, with pH changes critically controlling drug release and enhancing antitumor effects [27, 122]. Leveraging the vascular leakage phenomenon in tumor tissues, nanomaterials achieve passive targeting through the EPR effect. Active targeting is achieved by modifying nanocarriers with receptors, antibodies, and folic acid. Photothermal (PTT) and photodynamic therapies (PDT) play vital roles, with agents like AuNBP-Gd_2_O_3_/Au-dBSA under 808 nm near-infrared laser irradiation and Pheophorbide A under 660 nm laser irradiation significantly inhibiting tumor growth by generating ROS, inducing oxidative stress and cellular damage [33, 35]. Additionally, immunomodulatory nanopreparations, such as FEM@PFC, enhance immunogenic cell death and dendritic cell maturation, promote effector T cell activation, and curb regulatory T cell-mediated immunosuppression during radiation therapy [123]. Collectively, these strategies enable effective cancer therapy through targeted delivery, controlled release, PTT, and PDT, culminating in tumor proliferation inhibition, apoptosis induction, and improved tumor microenvironment.

Nanomedicine revolutionizes cancer therapy by addressing the limitations of traditional treatments like surgery, chemotherapy, and radiation therapy. These traditional methods often suffer from non-specificity, side effects, drug resistance, and inefficiency in treating metastatic cancers [6]. Nanomedicine employs various nanomaterials, encompassing metallic nanoparticles, liposomes, dendrimers, and quantum dots (QDs), to propel cancer therapy through targeted drug delivery, controlled release, and photothermal and photodynamic therapies [7]. For instance, QDs and metallic nanoparticles utilize their optical properties to enhance tumor detection and characterization, facilitating precise targeting of tumor cells and minimizing off-target effects [8, 9]. Controlled release mechanisms maintain drug activity at the tumor site, improving therapeutic outcomes. The distinct physical, chemical, and biological properties of nanomedicine facilitate a multifaceted approach to cancer therapy, including enhanced drug solubility and delivery efficiency, as well as superior diagnostic capabilities through imaging techniques [10] (Fig. 2). Collectively, these strategies underscore nanomedicine’s potential to provide more effective, targeted, and personalized cancer treatments (Table 1).

**Table 1:** Properties of different nanomaterials in cancer therapy.

Nanodrugs	Nanomaterials	Cancer cells	Function	Active/passive
TP-PMs [115]	Polymeric micelles	HT29	Induce apoptosis	Passive
TP/Ce6-LP + L [116]	Liposomes	HCC cells	Inhibit HCC progression	Passive
Apt-NPs-DTX [117]	Polymeric nanoparticle	CT26	Enhanced CT26 killing CC cells	Passive
C16-N/T hydrogel [118]	Nanogels	HCC cells	Restrain tumor proliferation	Passive
TPL@nano-gel [114]	Nanogels	MCF-7and MDA-MB-231	Inhibit antiangiogenic capacity	Passive
TP/Curc-NPs [119]	Polymeric nanoparticles	SKOV-3	Reduce triptolide-induced toxicity	Passive
PTX/TP-LPN [36]	Polymeric nanoparticles	NSCLC cells	Synergistic effect on lung cancer xenografts	Passive
TPL/NPs [120]	Polymeric nanoparticles	MCF-7and MDA-MB-231	Induce apoptosis/ Inhibit the expression of matrix metalloproteinases	Active
GC-TP-NPs [38]	Polymeric nanoparticles	HCC cells	Block TNF/NF-κB/BCL2 signaling	Active
Au-Dox [121]	Metallic nanoparticle	Cancer cells	Reduce cancer vitality	Passive
^177^Lu-BN-PLGA (PTX) [122]	Polymeric nanoparticles	MDA-MB-231	Facilitate paclitaxel delivery system	Passive
DOXs@BSA NPs [27]	Polymeric nanoparticles	Cancer cells	Improve serum stability	Passive
FEM@PFC [123]	Polymeric nanoparticles	Cancer cells	Immunosuppression and redox balance in TME	Passive
PPy-Te NPs [37]	Polymeric nanoparticles	Cancer cells	Improve biocompatibility	Passive
AuNRs@SiO_2_-RB@MnO_2_ [34]	Msetallic nanoparticles	Cancer cells	Improve the accuracy of tumor imaging	Passive

### Targeted drug delivery systems of nanomedicine

Targeted drug delivery systems (TDDS) offers a sophisticated approach to improving the precision and effectiveness of cancer treatments. By harnessing nanotechnology, these systems enable the precise delivery of therapeutic agents to tumor sites, minimizing systemic side effects and optimizing therapeutic outcomes [11]. TDDS operates through two main mechanism: passive and active targeting. Passive targeting exploits the unique physiological characteristics of tumors, such as the enhanced permeability and retention (EPR) resulting from their leaky vasculature [12]. This allows nanoparticles to accumulate passively within the tumor microenvironment, where they gradually release their therapeutic payload. Conversely, active targeting entails customing nanoparticle surfaces with specific ligands, including antibodies, peptides, or small molecules that bind to receptors overexpressed on cancer cells [13–15]. Active targeting improves the specificity of the delivery system and reduces off-target effects, which is particularly advantageous for tumors with limited EPR effect or when intracellular delivery of therapeutics is necessary.

Advanced therapeutic approaches integrated into TDDS include nuclear-targeted phototherapy nanodrugs, mitochondrial-targeted nanomedicines, multimodal imaging nanodrugs, and carrier-free nanodrugs. Nuclear-targeted phototherapy nanodrugs are designed to transport agents that generate reactive oxygen species (ROS) and induce hyperthermia within the cell nucleus upon light activation, efficiently disrupting DNA structures and selectively destroying cancer cells [16]. Mitochondrial-targeted nanodrugs enhance drug delivery to the mitochondria, amplifying therapeutic effects while minimizing collateral damage to healthy cells. Multimodal imaging nanodrugs carry diverse agents like fluorescent dyes and magnetic nanoparticles, providing high-resolution, real-time insights into tumor dynamics [17]. Carrier-free nanodrugs simplify drug delivery, increasing bioavailability and targeting precision [18].

The design and development of TDDS involve various nanocarriers, including liposomes, polymeric nanoparticles, dendrimers, and metallic nanoparticles [19–22]. Each carrier offers distinct advantages, such as biocompatibility, drug loading capacity, and potential for surface modification. Liposomes, with their phospholipid bilayer structure, have the capability to encapsulate both hydrophilic and hydrophobic drugs, rendering them highly versatile for active targeting strategies. For example, functionalized nano-sized liposomes equipped with anti-CD44 and anti-PD-L1 DNA aptamers were engineered to selectively target breast cancer cells. These liposomes carry Doxorubicin (DOX) and *IDO1* siRNA, promoting immunogenic cell death and inhibiting *IDO1* expression. This dual action effectively disrupts the tumor immune microenvironment (TIME), demonstrating notable antitumor efficacy in a subcutaneous breast cancer mouse model [23]. Polymeric nanoparticles are engineered for controlled drug release, responding to specific stimuli in the tumor microenvironment for precise drug delivery and regulate gene expression [24]. These advancements underscore the pivotal role of nanomedicine in developing more efficient and less toxic treatment modalitie. They offer a promising future where cancer treatment is not only more effective but also customized to minimize adverse effects on patients’ health.

### Controlled release mechanisms in nanomedicine

Controlled release mechanisms play a crucial role in optimizing cancer therapy efficacy within nanomedicine. These mechanisms leverage stimulus-responsive nanocarriers capable of reacting to diverse cues including heat, radiation, magnetic fields, and internal factors like pH and hypoxia [25]. This represents a significant advancement in cancer treatment.

Nanocarriers for controlled release are tailored to respond to the unique conditions of the tumor microenvironment. For example, a novel pH and thermal responsive carrier combines doxorubicin-loaded gold core-silica shell nanorods with salicylic acid-loaded poly (lactic-co-glycolic acid) based microspheres (NIMPS). Near-infrared laser irradiation or exposure to acidic environments can induce drug and nanorod release. This system exhibits excellent biocompatibility and is readily taken up by HeLa cells, facilitating localized combined therapy for cervical cancer cells [26]. Furthermore, research has developed a stimuli-responsive drug delivery mechanism by modifying bovine serum albumin (BSA) to create nanoparticles capable of controlled release. DOX-loaded BSA NPs were synthesized using a desolvation technique, further stabilized by crosslinking via Schiff base reactions, resulting in a pH-sensitive DOX delivery system (DOXs@BSA NPs) [27]. Coupling β-cyclodextrin (β-CD) with azobenzene-modified UiO-68 nanoparticles (β-CDcapped UiO-68-azo) creates a light-responsive or competitive binder-responsive release system. Additionally, stimuli-responsive metal-organic frameworks (MOFs) with azobenzene units on their surface can form supramolecular complexes with β-CD. Constructing stimuli-responsive MOFs is achieved by rinsing UiO-68-azo loaded with cargo through a β-CD aqueous solution, preventing premature release. This supramolecular complex can disassociate under various external stimuli, enabling controlled release of the cargo within the mechanized MOFs [28]. CURMs inhibit the growth of adenocarcinoma human alveolar basal epithelial cells and human umbilical vein endothelial cells [29]. These studies provide a solid foundation for the development of nanotechnology-enabled controlled release systems.

The controlled release of therapeutic agents, including systemic toxicity by limiting exposure to non-target tissues, enhancing patient compliance through reduced dosing frequency, and improving therapeutic outcomes by maintaining drug concentrations within a therapeutic window over time. However, challenges remain in maintaining nanocarrier stability during transit to target sites and achieving precise release kinetics.

### Photothermal and photodynamic therapies aligned nanomedicine in cancer treatment

Photothermal and photodynamic therapies are cutting-edge nanomedicine strategies for cancer therapy, employing light-sensitive nanoparticles to induce targeted tumor destruction [30, 31]. For instance, the multifunctional nanoplatform GNR-HA-ALA/Cy7.5-HER2, responsive to pH, glutathione (GSH), and hyaluronidase (HAase), has been engineered for precise breast cancer therapy. Demonstrating robust stability in blood circulation, this nanoplatform enables fluorescence imaging-guided combined PDT and PTT, resulting in significantly enhanced therapeutic efficacy compared to PDT or PTT alone. Thus, GNR-HA-ALA/Cy7.5-HER2 emerges as a promising candidate for treating HER2-positive breast cancer [32]. Another breakthrough theranostic nanoplatform, Au nanobipyramids with Gd2O3, Au nanoclusters, and denatured bovine serum albumin (AuNBP-Gd2O3/Au-dBSA), is tailored for FL/MR dual-modal imaging-guided photothermal therapy [33]. This platform exhibits exceptional photothermal anticancer effectiveness (over 95%) and low toxicity in both in vitro and in vivo studies.

PTT utilizes nanoparticles capable of absorbing near-infrared (NIR) light and converting it into heat, resulting to localized hyperthermia that effectively eradicates tumor cells. The NIR region is preferred for its superior tissue penetration and minimal absorption by biological tissues, enabling precise heating of the tumor site while sparing adjacent healthy cells. Nanoparticles commonly employed in PTT, such as gold nanorods, gold nanoshells, and copper sulfide nanoparticles, exhibit robust absorption in the NIR spectrum, rendering them highly efficient for this purpose. Recent research introduced AuNRs@SiO_2_-RB@MnO_2_, a novel nanotheranostic, which encapsulates gold nanorods within a silica layer for NIR-II light absorption and MnO_2_ nanosheet adsorption [34]. This design enables dual-mode chemodynamic and photothermal cancer therapy via Fenton-like reactions and heat generation, while its fluorescence recovery signals glutathione consumption for effective photoacoustic imaging-guided treatment. The precise control over nanoparticle distribution and the laser parameters enables targeted tumor ablation with minimal side effects, presenting a promising strategy for treating challenging tumors unresponsive to conventional methods.

In contrast, PDT employs photosensitizing agents that, upon activation by light of a specific wavelength, generate ROS capable of damaging and killing cancer cells [35]. Nanoparticles play a crucial role in PDT by facilitating the delivery and localization of photosensitizers to the tumor site, enhancing the generation of ROS, and minimizing systemic toxicity. Commonly used photosensitizers can be encapsulated or conjugated to nanoparticles, which can then be further functionalized to specifically target tumor cells. A study outlined the development of a ROS self-activatable nanosystem, PTKPa, which incorporates poly(thioketal) linked with pheophorbide A (Ppa) photosensitizers on its side chains. This design aims to mitigate aggregation-caused quenching and enhance PDT efficiency. Upon laser irradiation, ROS generated from PTKPa catalyzes the breakdown of poly(thioketal), releasing Ppa and generating additional ROS. This process enhances PDT’s effectiveness via a feedback loop that amplifies oxidative stress, resulting in tumor cell destruction and immunogenic cell death [35]. The activation of photosensitizers by light exposure results in the generation of singlet oxygen and other ROS, capable of directly damaging tumor cells and vasculature, and inducing immunogenic cell death, fostering an antitumor immune response. PDT’s selectivity and ability to activate treatment at a specific time and location make it an attractive option for treating superficial tumors and those accessible via endoscopic techniques. Both PTT and PDT offer several advantages, including high precision, minimal invasiveness, and the potential for synergistic effects when combined with other cancer treatments. However, challenges persist, including ensuring adequate light penetration for deep tumors and avoiding nonspecific activation of photosensitizers or heat generation.

Taken together, nanomedicine’s properties are fundamentally determined by the design of TDDS, which are crucial for their functionality and application [11, 36]. Utilizing appropriate nanocarriers allows these nanomaterials to selectively target cancer cells while minimizing damage to normal cells [21, 24, 37]. Controlled release mechanisms, integral to the TDDS design, ensure precise dosages and the effective release of both therapeutic agents and nanocarriers. Additionally, PDT and PDD rely on these controlled release properties [16, 37, 38]. By integrating TDDS, controlled release, and phototherapy, nanomedicine can precisely target cancer cells, providing innovative and targeted therapeutic interventions. Therefore, in clinical applications, adopting suitable design strategies tailored to specific therapeutic requirements is essential.

## Nanomedicine in regulating PCD in cancer cells

**Fig. 3:**
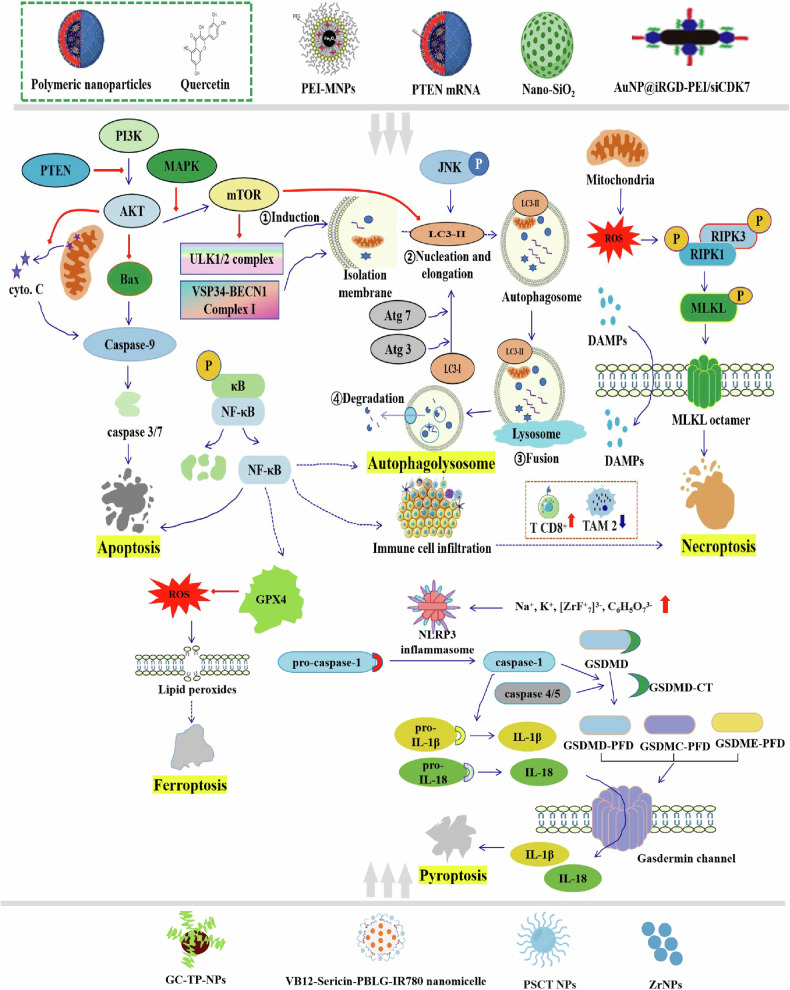
Nanomedicine in regulating PCD in cancer cells. Nanomedicine induce various forms of PCD, contributing to their antitumor effects. Primarily, they inhibit the PI3K-AKT axis, prompting the release of cytochrome C and upregulation of caspase cascade proteins to trigger apoptosis [125]. Secondly, they modulate autophagy by either suppressing the PI3K/AKT/mTOR pathway or activating the MAPK/mTOR/ULK1 pathway, with JNK signaling also participating in core formation and elongation [128, 129, 132]. Additionally, mitochondrial-produced ROS induce cell death via the RIPK1/RIPK3/MLKL pathway, while released damage-associated molecular patterns (DAMPs) activate immune responses, exemplified by the AuNP/siCDK7 system which boosts CD8^+^ T cell infiltration and diminishes M2 macrophage presence, thus improving the immunosuppressive microenvironment and enhancing anti-PD-1 therapy efficacy [69, 134]. Furthermore, nanomedicines suppress the NF-κB pathway, downregulate antioxidant enzyme GPX4, induce ROS accumulation, and trigger lipid peroxidation-mediated ferroptosis [38]. They also release significant quantities or elevate mitochondrial ROS in cancer cells, activating the NLRP3 inflammasome and inducing pyroptosis via the NLRP3/Caspase-1/gasdermin D pathway, as seen in PSCT NPs [89], VB12-Sericin-PBLG-IR780 nanomicelle [142], and ZrNPs [143]. Collectively, these mechanisms modulate various signaling pathways such as PI3K/AKT/mTOR, NF-κB, JNK, and NLRP3/Caspase-1/GSDMD to exert antitumor effects.

Nanomedicine significantly influences PCD within cancer cells, employing a multifaceted approach to trigger various cellular processes and pathways leading to cell death (Table 2). These mechanisms of action encompass both direct interventions on cancer cells and indirect modulation of the tumor microenvironment, potentially leading to apoptosis, autophagy, or necroptosis. Nanomedicine provides a potent strategy to induce the destruction of cancer cells including those resistant to traditional therapies, by directly targeting the cellular and molecular pathways controlling cell death (Fig. 3).

**Table 2:** Nanomaterials Regulating PCD in cancer therapy.

Nanomaterials	Types of PCD	Target	Receptor	Pathways	Related cytokines
NREA [124]	Apoptosis	G2/M cell	/	JKN	c-Myc BRD4
PLGA-QNPs [125]	Apoptosis	Cervical cancer cells	/	PI3K/AKT Suppression	Caspase-3,7
Graphene oxide [126]	Apoptosis	Colorectal cancer	/	AMPK/mTOR/ULK-1	/
LYC-NPs [127]	Apoptosis	HepG2 (Hepatocellular carcinoma)	/	Matrix metalloproteinase-2 (MMP-2) and MMP-9	/
MNPs [128]	Autophagy	Cervical cancer	/	mTOR-Akt-p70S6 K ATG7 NF-κB TGF-β	/
Graphene oxide (GO) [129]	Autophagy	Cancer cells	Folate receptor	JNK/p53/p21	MTH1 ICG
Liposomes [130]	Autophagy	CAF	TH integrin	Aerobic glycolysis	TR
NBP/TiO_2_ [131]	Autophagy	U-87 MG cell	/	/	/
PTEN mRNA nanoparticles [132]	Autophagy	Cancer cells	Anti-programmed death-1 antibody	PI3K/Akt/mTOR	IL-12; TNF-α; IFN-γ
iron oxide NPs [133]	Autophagy	A549 cells	/	AMPK -mTOR-AKT	/
Nano-SiO_2_ [134]	Necroptosis	HepG2	/	RIPK1/RIPK2/MLKL	/
SeNPs [135]	Necroptosis	PC-3 (prostate adenocarcinoma cells)	/	/	TNF; IRF-1
N-TiO_2_ NPs [136]	Necroptosis	A375 cells (human melanoma)	/	/	/
CuS-NiS_2_ [137]	Necroptosis	Gastric cancer	/	MLKL/CAPG	/
Ferumoxytol [138]	Ferroptosis	Breast cancer cells	TLR3	NF-κB	PIC TAM MAPK Syk
Ferumoxytol [139]	Ferroptosis	Prostate cancer	ULBP	PD-L1 HMGB1	IFN-γ
manganese-coordinated nanomedicine [140, 141]	Pyroptosis	Tumor endothelial cell	PD1 CTLA4	cGAS-STING	cGAMP
VB12-Sericin-PBLG-IR780 Nanomicelles [142]	Pyroptosis	ATP5MC3	/	NLRP3/Caspase-1/gasdermin D	/
PSCT NPs [89]	Pyroptosis	Tumor cells	/	Caspase-1/gasdermin D & Caspase-8/gasdermin C	/
ZrNPs [143]	Pyroptosis	Lung cancer	/	Gasdermin D	IL-1β Caspase-1

### Nanotechnology-driven apoptosis in cancer cells

Apoptosis plays a crucial role in eliminating cancer cells. This involves the activation of caspases, cysteine-aspartic proteases that orchestrate cell dismantling by cleaving specific substrates. This leads to hallmark morphological changes, including chromatin condensation, DNA fragmentation, and membrane collapse [39, 40]. Ultimately, this process results in the phagocytic removal of cell remnants [41], preventing inflammatory responses. Regulatory proteins, including the *Bcl-2* family, inhibitor of apoptosis proteins (IAPs), and the tumor suppressor *p53*, meticulously govern this complex sequence [42]. They balance cell survival and death in response to physiological and pathological cues, highlighting apoptosis’s essential role in development, immune function, and disease prevention [43, 44]. Essentially, apoptosis relies on a cascade of enzymatic activities (Fig. 3). Thus, the intersection between apoptosis and nanomedicine is also based on these enzymatic processes.

Nanoparticles can be engineered to induce apoptosis directly by delivering pro-apoptotic drugs or genes specifically to cancer cells. For instance, nanoparticles conjugated with ligands targeting death receptors on the surface of cancer cells can activate the extrinsic apoptotic pathway [45]. Likewise, nanoparticles designed to release cytochrome c inside the cells can activate the intrinsic mitochondrial pathway, leading to apoptosis [46]. This targeted approach ensures selective induction of apoptosis in cancer cells, sparing healthy cells, and reducing side effects. Nanocarriers can encapsulate and deliver chemotherapeutic agents capable of inducing apoptosis through either the intrinsic (mitochondrial) or extrinsic (death receptor) pathways [47, 48]. Research indicates that co-delivering DOX and erlotinib with transferrin-modified liposomal nanoparticles significantly enhances glioblastoma treatment [49]. Additionally, a study on *Rhynchosia fairholmianus* capped zinc oxide nanoparticles (RFZnO NPs) revealed a decrease in cell proliferation and an increase in cytotoxicity and ROS. Importantly, this study observed a significant increase in apoptosis markers, including *cytochrome c* and caspase 3/7, along with higher levels of pro-apoptotic proteins (p53, Bax), and a decrease in the anti-apoptotic protein Bcl-2 [50]. Similarly, a study investigated a noninvasive chemo-photodynamic therapy using polyethylene glycol-coated zinc oxide nanorods (PEG-ZnO NRs) and piperlongumine (PL) for targeted cancer treatment [51]. To enhance ROS generation, the nanorods were modified with gold nanoparticles (AuNPs), resulting in even higher ROS yields and cytotoxicity against cancer cells upon exposed to UV light. Alternatively, activation of the extrinsic pathway can facilitate the binding of ligands to death receptors on cancer cells, triggering cell death. This dual-pathway approach expands the therapeutic potential, providing personalized treatment strategies for different types of cancer.

In conclusion, this targeted delivery mechanism in inducing apoptosis enhances chemotherapy efficacy by ensuring the direct drug release to the affected cells while minimizing systemic toxicity, thus improving patient outcomes. By leveraging the intrinsic pathway, nanocarriers facilitate the release of cytochrome c from mitochondria, initiating apoptosis.

### Autophagy regulation through nanomedical interventions

Autophagy, the process by which cells digest their own components, can exert dual roles in cancer, either promoting survival or inducing cell death [52]. The regulation of autophagy via nanomedical interventions has emerged as a promising therapeutic approach for diverse diseases such as cancer, neurodegeneration, and infectious diseases [53]. Nanomedicine can potentially modulate autophagy pathways with precision, enabling either promotion or inhibition of this process, thus providing opportunities for therapeutic and cytoprotective interventions (Fig. 3).

Nanomedicine can modulate autophagy to induce cancer cell death by delivering molecules that regulate the autophagic process. For instance, nanoparticles loaded with siRNA or drugs targeting autophagy-related genes can drive cancer cells toward apoptosis [54, 55]. Nanoparticles can drive cancer cells toward autophagic cell death by delivering drugs that either upregulate autophagy or inhibit its suppression. Intriguingly, studies have shown that cell-penetrating nanoparticles, specifically of different sizes, differentially activate immune pathways. Larger gold (Au) nanoparticles, greater than 10 nm, initiate the NF-κB signaling pathway, while ultrasmall gold nanoparticles, less than 10 nm, selectively activate the NLRP3 inflammasome, resulting in Caspase-1 maturation and interleukin-1β production. This implies that Au4.5 nanoparticles mitigate the LC3-mediated inhibition of the NLRP3 inflammasome by promoting LC3 degradation [56]. In vitro studies demonstrated that gold-coated mesoporous silica nanoparticles (GCMSNs) induced more significant oxidative stress in lung cancer cells (A549) compared to normal cells (3T3-L1). This oxidative stress led to mitochondrial dysfunction, primarily contributing to mitochondria-mediated autophagy, thus inhibiting cancer cell growth [57]. The study strategically incorporated the respiration inhibitor 3-bromopyruvate (3BP), known for inducing autophagy and mitigating hypoxia, into chlorin e6 (Ce6)-encapsulated nanoparticles for the treatment of hypoxic tumors. 3BP significantly decreased intracellular oxygen consumption by downregulating hexokinase-II (HK-II) and glyceraldehyde 3-phosphate dehydrogenase (GAPDH) expression, consequently alleviating tumor hypoxia and enhancing the efficacy of PDT [58]. The design of these nanomedicines aims to induce both autophagy and apoptosis simultaneously, leveraging the interplay between these pathways to effectively eradicate cancer cells.

In addition to the aforementioned aspects, the intricate interplay between methylation processes and autophagy within the context of DNA and RNA modifications in cancer has recently become a significant focus of research [59]. Nevertheless, the potential of nanomedicine to serve as modulators for DNA and RNA remains ambiguous, underscoring the need for extensive research to gain a comprehensive understanding of their role in the broader system.

### Necroptosis as a target for nanomedicine in cancer therapy

Necroptosis, a form of programmed necrosis, serves as an alternative cell death pathway in cancer cells, particularly those resistant to apoptosis [60]. Nanoparticles have the capability to induce necroptosis by delivering molecules that activate the signaling pathways associated with this form of cell death [61]. Nanomedicines can initiate a cascade leading to the rupture of cancer cell membranes and subsequent cell death by targeting specific receptors or signaling proteins involved in necroptosis, such as RIPK1 or RIPK3 (Fig. 3) [62].

From an immunological perspective, the strategic introduction of necroptotic cells into the tumor microenvironment is observed to enhance BATF^3+^cDC1^−^ and CD8^+^ leukocyte-mediated antitumor immunity [63]. This enhancement is characterized by a significant increase in the presentation of tumor antigens by tumor-associated antigen-presenting cells, highlighting the efficacy of necroptotic cells in boosting antitumor immunity. Additionally, investigations highlight the activation of RIPK1/RIPK3 as a critical upstream target that facilitates the initiation of tumor immunity, indicating its potential therapeutic potential in cancer treatment strategies [64]. Furthermore, eliminating RIPK3 decreased lung tumor nodules by 46%, while inhibition of RIPK1’s kinase activity achieved a 38%. However, altering RIPK3’s kinase activity or MLKL had minimal effect on lung tumor formation (Fig. 3) [65]. The reduction in tumor nodules upon RIPK3 deletion was primarily associated with the stromal rather than the hematopoietic compartment, emphasizing the significance of the tumor microenvironment in cancer progression.

*CDK7* expression is frequently dysregulated in cancer, contributing to oncogenesis by promoting uncontrolled cell proliferation and survival [66]. The development of nanoparticle-based strategies for *CDK7* targeting offers promising avenues for cancer therapy [67]. Nanoparticles, like gold nanoparticles conjugated with internalizing-RGD (iRGD), can be engineered to deliver siRNA targeting *CDK7* (*siCDK7*) directly to the tumor microenvironment [68]. Such targeted delivery system ensures high specificity and efficiency in silencing *CDK7*, effectively inhibiting tumor growth and progression. The study presented a sophisticated approach utilizing a gold nanoparticle (AuNP) system conjugated with internalizing-RGD (iRGD) and encapsulating siRNA targeted at *CDK7* [69]. This innovative AuNP/si*CDK7* system demonstrated remarkable precision in tumor targeting and exhibited notable photothermal capabilities. The strategic implementation of this system brought about significant transformation in the tumor microenvironment. It alleviated immunosuppression by reducing M2 macrophage presence and significantly enhanced CD8+ T cell infiltration [70, 71]. This strategic reconfiguration of the tumor environment substantially enhanced the efficacy of anti-PD-1 therapy, showcasing the system’s dual functionality in directly combating tumor cells and modulating the immune landscape to bolster a more robust antitumor response.

### Nano-enabled immunogenic cell death in cancer treatment

Immunogenic cell death is a form of PCD that not only eliminates cancer cells but also stimulates an immune response against the tumor [4, 72]. Immunomodulatory nanomedicines targeting cancer cells aim to induce ICD, thereby enhancing the cancer-immunity cycle, reducing systemic (lymphocyte) toxicity, and improving antitumor immunity and immunotherapy outcomes [73]. The effectiveness of anticancer immunotherapy relies on nanomedicines that modulate the TIME. TIME often has elevated levels of immunosuppressive pathways and mediators. These drugs counteract immunosuppression, enhancing the presence, proliferation, and effectiveness of cytotoxic T cells at the tumor site, thereby improving local and systemic antitumor immunotherapy outcomes [74]. To address this issue, nanoparticles can deliver checkpoint inhibitors, such as anti-PD-1 or anti-CTLA-4 antibodies, directly to the tumor microenvironment [75, 76]. Nanomedicines targeting the peripheral immune system, encompassing secondary lymphoid organs, lymph nodes (LN), and the spleen, can substantially enhance antigen presentation, cytotoxic T cell generation, and T cell vitality and activity, providing a comprehensive approach to enhancing cancer treatment efficacy [77].

Nanoparticles can induce ICD by releasing of chemotherapeutic agents that trigger the emission of DAMPs from dying cancer cells. These DAMPs then stimulate the immune system to recognize and destroy remaining cancer cells [78]. Nanomedicine’s ability to precisely control ICD induction presents a promising approach for combining direct cancer cell killing with immunotherapy. The mechanistic foundation of ICD involves the release of DAMPs from dying cancer cells. DAMPs such as calreticulin (CRT), ATP, and high-mobility group box 1 (HMGB1) act as critical signals for recruiting and activating dendritic cells (DCs). CRT relocation to the external plasma membrane leaflet serves as a critical signal for dendritic cell-mediated phagocytosis of tumor cells. Additionally, ATP acts as a chemotactic factor, drawing immune cells into the tumor microenvironment. Moreover, HMGB1 passively released from necrotic cells, binds to toll-like receptor 4 (TLR4) on DCs, facilitates their maturation and the effective presentation of tumor antigens to T cells. Nanoparticles, designed to encapsulate ICD-inducing agents like chemotherapeutic drugs, photosensitizers for PDT, or radiotherapy agents, significantly enhance the precision and effectiveness of cancer therapies. Deploying these nanoparticles ensures localized and controlled induction of ICD within the TIME, minimizing systemic toxicity while enhancing antitumor immunity [78–80]. Researchers are exploring how nanoparticles can be designed not only to deliver ICD-inducing agents but also directly stimulate the immune system. For example, recent studies have focused on nanoparticles capable of delivering CpG oligodeoxynucleotides, a potent TLR9 agonist, alongside ICD-inducing agents to synergistically promote dendritic cell maturation and T cell activation [81–83]. Additionally, the use of nanoparticles to co-deliver ICD-inducing agents and immune checkpoint inhibitors, such as PD-L1 antibodies, with the goal of both kill cancer cells in an immunogenic manner and enhancing the overall immune response against the tumor [84].

By leveraging the specificity and controlled release capabilities of nanoparticles, researchers can deliver ICD-inducing agents directly to the tumor microenvironment. The ongoing research and development in nanoparticle-mediated ICD emphasize the crucial convergence of nanotechnology, immunology, and oncology, envisioning a future where cancer treatment involves both leveraging the body’s defenses and targeting the disease.

### Other PCD in cancer treatment

While apoptosis, atuophagy and necroptosis have been the primary focus of cancer research and therapy, the growing understanding of alternative cell death pathways, including pyroptosis, ferroptosis and entosis, is reshaping our approach to cancer treatment. Pyroptosis and ferroptosis, in particular, are distinct yet equally significant pathways of cell death [85].

Pyroptosis, characterized by gasdermin-mediated pore formation leading to cell lysis and robust inflammatory responses, provides a distinct pathway for enhancing antitumor immunity [86]. It commences with the recognition of danger signals by cellular pattern recognition receptors (PRRs), including pathogen-associated molecular patterns (PAMPs) and DAMPs. Upon recognition, these PRRs instigate the formation of inflammasomes, consisting of sensor proteins like NLRP3, ASC, and pro-caspase-1. These inflammasomes serve as platforms facilitating the conversion of pro-caspase-1 into its active form, caspase-1, which subsequently cleaves gasdermin D. The cleaved fragments of gasdermin D then oligomerize and integrate into the plasma membrane, creating pores that disrupt cellular ion gradients, leading to osmotic swelling, membrane rupture, and ultimately cell lysis (Fig. 3). In one study, the modulation of mitochondria inducing osteosarcoma pyroptosis, through inhibiting the pyruvate dehydrogenase kinase 1 (PDHK1), was achieved by the polymer micelle of dichloroacetate, which promotes enhanced antitumor efficacy in combination with immunotherapy [87]. Meanwhile, BMS-202 can effectively block the immune escape mediated by upregulation of *PD-L1* expression caused by increased IFN-γ secretion after PIT, further amplifying the effect of PIT [88]. Numerous investigations have uncovered the intricate relationship between pyroptosis activation and the enhancement of cancer immunotherapy, providing novel perspectives in multi-PCD therapy [89–91].

Ferroptosis, driven by iron-dependent lipid peroxidation, exploits metabolic vulnerabilities in cancer cells, offering a novel therapeutic approach [92]. In contrast to apoptosis or necrosis, ferroptosis is specifically induced by the accumulation of lipid ROS and is significantly impacted by the cell’s iron metabolism. The process occurs through a tightly regulated series of steps, underscoring the crucial equilibrium of iron, antioxidants, and lipid peroxides within the cell. Therefore, ferroptosis therapy has recently emerged as a novel approach in cancer treatment. Studies demonstrate that ferroptosis can be enhanced by accelerating the Fenton reaction. Ferroptosis is initiated when either cysteine availability is compromised, resulting in reduced GSH synthesis, or when GPX4 activity is directly inhibited (Fig. 3). This leads to an uncontrolled accumulation of lipid ROS, particularly lipid hydroperoxides, serving as a catalyst for the Fenton reaction. Fe^2+^ reacts with hydrogen peroxide to produce highly reactive hydroxyl radicals, thereby intensifying lipid peroxidation and cellular damage. Notably, a combination strategy involving cisplatin (CDDP)-loaded Fe3O4/Gd_2_O_3_ hybrid nanoparticle, along with lactoferrin (LF) and RGD dimer (RGD2), forming the FeGd-HN@Pt@LF/RGD2, demonstrates the ability to penetrate the blood-brain barrier. Consequently, this formulation releases Fe^2+^, Fe^3+^, and CDDP upon endosomal uptake and degradation within cancer cells [93]. The DAR nanoactivator, consisting of DOX, Tannic Acid, and the photosensitizer IR820, demonstrates a novel anticancer mechanism through internalization by tumor cells and activation by protons within acidic lysosomes [94, 95]. It effectively establishes an intracellular positive feedback loop driven by endogenous iron release. Successful activation of immune therapy recruits CD8 T cells to the tumor site, enhancing tumor cell ferroptosis via IFNγ-related pathways, representing a significant advancement in efficient tumor eradication strategies.

Mitotic catastrophe, arising from mitotic errors, capitalizes on the unbridled proliferation of cancer cells, offering an alternative route to cell death distinct from classical apoptosis and providing insights into drug resistance [96, 97]. The onset of mitotic catastrophe is intricately tied to the integrity of cell cycle checkpoints, particularly the G2/M checkpoint, which verifies that cells with fully replicated and undamaged DNA advance to mitosis. Cells with compromised genomic integrity improperly entering mitosis exhibit pronounced chromosomal instability, leading to misaligned chromosomes, defects in spindle assembly, and erroneous chromosome segregation. Mitotic catastrophe’s conclusion can result in various forms of cell death, such as apoptosis, necrosis, or cellular senescence, frequently characterized by morphological alterations like micronucleation or multinucleation. Due to its role in preserving genomic stability, mitotic catastrophe is a focal point in cancer therapy, with numerous anticancer agents aimed at inducing this pathway through DNA damage or disruption of spindle assembly. Cell Division Cycle 5-Like (Cdc5L) functions as a regulatory factor in the mitotic process, influencing the splicing expression of pre-messenger RNA (pre-mRNA) for genes involved in mitosis and the DNA damage response. Inhibition or absence of *CDC5L* can impede mitosis progression and lead to mitotic mutations. Notably, *CDC5L* exhibits high expression levels in cervical tumors and osteosarcomas, underscoring its potential as a target for cancer therapy [98]. Furthermore, the substantial overexpression of *CDC5L* in cervical tumors, bladder cancer, gliomas, and osteosarcomas emphasizes its critical role in the pathogenesis of these cancers and its potential as a therapeutic target.

Moreover, entosis, a process involving the engulfment of one cell by another, underscores the complexity of cell death mechanisms amenable to nanotechnological interventions [99, 100]. The mechanism governing entosis is tightly controlled by the RhoA-ROCK signaling pathway, critical for remodeling the actin cytoskeleton necessary for cell motility and morphological changes. Additionally, the process is influenced by the expression levels of E-cadherin, a cell adhesion molecule facilitating the initial recognition and adhesion between cells during the entosis phase. For example, a study revealed the mechanism of action of cell entosis in mitotic surveillance. This process selectively promotes the engulfment of aneuploid progeny cells into neighboring cells, forming cell-in-cell structures through activation of the p53 signaling pathway, which eliminates potentially tumorigenic cells and preserves epithelial genomic stability [101]. Nanoformulations facilitate efficient internalization, drug delivery, and inactivation of cancer cell, including cancer stem cells (CSC) from patient-derived samples, making them promising therapeutic agent [102]. Additionally, research indicates that DNA-AuNPs, created by mixing calf thymus DNA with HAuCl4 act as radiosensitizers for human glioma cells with CSC-like characteristics, reducing their survival rate [103]. This occurred not through induction of ROS or apoptosis but by increasing abnormal cell nuclei with extensive γ-H2AX foci, indicating cell death via mitotic catastrophe. Thus, DNA-AuNPs show promise as anti-CSC radiosensitizing agents.

Collectively, these mechanisms highlight the intricate tactics employed by nanomedicine against cancer, expanding beyond conventional cell death pathways to leverage the complete spectrum of PCD.

## Discussion

Nanomedicine has revolutionized oncology, particularly strategically inducing PCD within cancer cells [104]. Leveraging the unique physicochemical properties of nanoscale materials, nanomedicine enables targeted delivery of therapeutic agents directly to tumor sites, enhancing treatment specificity and efficiency significantly. One of the foremost benefits of this methodology is the potential for precise targeting, minimizing off-target effects and reducing toxicity to healthy cells. Additionally, nanoparticles’ ability to encapsulate and protect therapeutic agents enhances their stability and bioavailability, addressing the rapid degradation and clearance issues associated with many conventional drugs.

Despite the promising advancements, challenges such as safety concerns, off-target effects, and biocompatibility issues present significant obstacles. Addressing these challenges necessitates meticulous nanoparticles design, ensuring biocompatibility, target specificity, and safe degradation post-treatment within the body [105]. Enhancing targeting specificity through the functionalization of nanoparticle surfaces with cancer-specific ligands or antibodies is crucial for reducing off-target effects. This approach ensures selective binding and internalization of nanoparticles by cancer cells, thereby minimizing unintended interactions with healthy tissues. Additionally, employing materials that are inherently non-toxic and compatible with the body, such as liposomes or biodegradable polymers, can improve the biocompatibility of nanocarriers, further safeguarding patient health. Focusing on these targeted strategies allows us to overcome the material-related limitations of PCD-based nanotherapy, paving the way for safer, more effective cancer treatments.

Tumor heterogeneity and the intricate tumor microenvironment further complicate the efficacy of PCD-based nanotherapies. Genetic and proteomic variations among patients and within tumors can result in inconsistent therapeutic responses and adverse effects. Using patient-derived cells as carriers for therapeutic agents presents a novel approach to enhance specificity and efficacy [106, 107]. Targeting epigenetic modifications involved in gene expression regulation through nanomedicine [59], such as methyltransferase-like (METTL), YTH domain family and FTO, emerges as a potential therapeutic approach [108, 109]. Hence, could nanomedicine attenuate further regulation? Customizing therapies according to a patient’s unique cancer profile enables navigating the complexities of tumor heterogeneity and microenvironmental factors, leading to more effective and less toxic.

Nanomedicine is pivotal in cancer diagnostics, using nanoscale dimensions, precision release, and targeted delivery for site-specific localization. Nanomaterials targeting prostate-specific membrane antigens (PSMA) and prostate-specific antigen (PSA) show promise in prostate cancer detection. Magnetic nanoparticles targeting PSMA enhance MRI contrast by congregating at tumor margins in mouse models [110]. Manganese oxide-mesoporous silica nanoparticles (Mn-MSNs) targeting PSA accumulate specifically in prostate cancer cells, improving imaging [111]. Targeted nanobubbles with anti-PSMA aptamer facilitate ultrasonic imaging for early prostate cancer detection [112]. Fluorescently tagged CD47 antibodies (anti-CD47) enhance bladder cancer cells identification, aiding diagnostic precision and surgical thoroughness [113]. These examples underscore the potential of nanomaterials in improving the accuracy and specificity of cancer diagnostics. Nevertheless, challenges such as sensitivity, specificity, and accurate monitoring of therapeutic efficacy and tumor response persist.

Nanomedicine plays a crucial role in modulating PCD for cancer treatment, offering significant advancements in improving patient outcomes. However, realizing the full potential of these innovative treatments requires a multidisciplinary approach, spanning material science, oncology, genomics, bioinformatics, and other fields. The clinical translation of nanomedicine-based PCD therapies necessitates rigorous research, development, and collaborative effort to ensure accessibility and benefits for all cancer patients, raising several critical questions. Can nanomaterials mediating PCD be engineered to serve a dual function, integrating detection and therapy of cancer cells [25, 33, 32]? Considering the biocompatibility issues of nanomaterials, is it feasible to fabricate personalized nanomedicines using tissues extracted from patients themselves [114]? Can nanotechnology provide innovative solutions for real-time monitoring of PCD progression in cancer cells during treatment [12, 17]? How can nanomedicine strategies induce PCD in cancer cells also prevent immune evasion, thus enhancing the immune system’s ability to recognize and destroy tumor cells [35, 74, 88]? What strategies can be employed to assess and ensure the long-term safety and efficacy of nanomedicines in cancer therapy? Can nanomedicine be leveraged to overcome multidrug resistance by targeting PCD pathways in cancer therapy? Addressing these questions will deepen our understanding of the crucial mechanisms by which nanomedicine regulates life activities in cancer cells.

Nanomedicine offers the promise of revolutionizing cancer treatment, providing hope for more effective and personalized therapeutic strategies in combating this pervasive disease. By addressing challenges and leveraging nanotechnology’s advantages, we can achieve the realization of more precise, side-effect-free, and effective cancer therapies.

## References

[1] Litwin MS, Tan HJ. The diagnosis and treatment of prostate cancer: a review. JAMA. 2017;317:2532–42. 28655021 10.1001/jama.2017.7248

[2] Wei G, Wang Y, Yang G, Wang Y, Ju R. Recent progress in nanomedicine for enhanced cancer chemotherapy. Theranostics 2021;11:6370–92. 33995663 10.7150/thno.57828 PMC8120226

[3] Björnmalm M, Thurecht KJ, Michael M, Scott AM, Caruso F. Bridging bio-nano science and cancer nanomedicine. ACS Nano. 2017;11:9594–613. 28926225 10.1021/acsnano.7b04855

[4] Meng Q, Ding B, Ma P, Lin J. Interrelation between programmed cell death and immunogenic cell death: take antitumor nanodrug as an example. Small Methods. 2023;7:e2201406. 36707416 10.1002/smtd.202201406

[5] Paulson KG, Voillet V, McAfee MS, Hunter DS, Wagener FD, Perdicchio M, et al. Acquired cancer resistance to combination immunotherapy from transcriptional loss of class I HLA. Nat. Commun. 2018;9:3868. 30250229 10.1038/s41467-018-06300-3 PMC6155241

[6] Fan D, Cao Y, Cao M, Wang Y, Cao Y, Gong T. Nanomedicine in cancer therapy. Signal Transduct. Target Ther. 2023;8:293. 37544972 10.1038/s41392-023-01536-y PMC10404590

[7] Cheng Z, Li M, Dey R, Chen Y. Nanomaterials for cancer therapy: current progress and perspectives. J Hematol Oncol. 2021;14:85. 34059100 10.1186/s13045-021-01096-0 PMC8165984

[8] Liu Y, Wang Y, Song S, Zhang H. Tumor diagnosis and therapy mediated by metal phosphorus-based nanomaterials. Adv Mater. 2021;33:e2103936. 34596931 10.1002/adma.202103936

[9] Wang Y, Tay A. Advances in enantiomer-dependent nanotherapeutics. ACS Nano. 2023;17:9850–69. 37267453 10.1021/acsnano.3c02798

[10] Ahmad A, Rashid S, Chaudhary AA, Alawam AS, Alghonaim MI, Raza SS, et al. Nanomedicine as potential cancer therapy via targeting dysregulated transcription factors. Semin Cancer Biol. 2023;89:38–60. 36669712 10.1016/j.semcancer.2023.01.002

[11] Wang K, Shen R, Meng T, Hu F, Yuan H. Nano-Drug Delivery Systems Based on Different Targeting Mechanisms in the Targeted Therapy of Colorectal Cancer. Molecules. 2022;27:2981. 35566331 10.3390/molecules27092981 PMC9099628

[12] Shi Y, van der Meel R, Chen X, Lammers T. The EPR effect and beyond: strategies to improve tumor targeting and cancer nanomedicine treatment efficacy. Theranostics. 2020;10:7921–4. 32685029 10.7150/thno.49577 PMC7359085

[13] Verhaar ER, Woodham AW, Ploegh HL. Nanobodies in cancer. Semin Immunol. 2021;52:101425. 33272897 10.1016/j.smim.2020.101425 PMC8164649

[14] Carter PJ, Rajpal A. Designing antibodies as therapeutics. Cell. 2022;185:2789–805. 35868279 10.1016/j.cell.2022.05.029

[15] Liu Y, Qian X, Ran C, Li L, Fu T, Su D, et al. Aptamer-based targeted protein degradation. ACS Nano. 2023;17:6150–64. 36942868 10.1021/acsnano.2c10379

[16] Long X, Zhang X, Chen Q, Liu M, Xiang Y, Yang Y, et al. Nucleus-targeting phototherapy nanodrugs for high-effective anti-cancer treatment. Front Pharm. 2022;13:905375. 10.3389/fphar.2022.905375 PMC9130747 35645841

[17] Gong X, Wang Z, Zhang L, Dong W, Wang R, Liu Y, et al. A novel carbon-nanodots-based theranostic nano-drug delivery system for mitochondria-targeted imaging and glutathione-activated delivering camptothecin. Colloids Surf B Biointerfaces. 2022;218:112712. 35921692 10.1016/j.colsurfb.2022.112712

[18] Huang L, Hu S, Fu YN, Wan Y, Li G, Wang X. Multicomponent carrier-free nanodrugs for cancer treatment. J Mater Chem B. 2022;10:9735–54. 36444567 10.1039/D2TB02025D

[19] Zhou H, Yu CY, Wei H. Liposome-based nanomedicine for immune checkpoint blocking therapy and combinatory cancer therapy. Int J Pharm. 2024;652:123818. 38253269 10.1016/j.ijpharm.2024.123818

[20] Danaeifar M, Negahdari B, Eslam HM, Zare H, Ghanaat M, Koushali SS, et al. Polymeric nanoparticles for DNA vaccine-based cancer immunotherapy: a review. Biotechnol Lett. 2023;45:1053–72. 37335426 10.1007/s10529-023-03383-x

[21] Pricl S. The Spicy Science of Dendrimers in the Realm of Cancer Nanomedicine: A Report from the COST Action CA17140 Nano2Clinic. Pharmaceutics. 2023;15:2013. 37514199 10.3390/pharmaceutics15072013 PMC10384593

[22] Subhan MA, Muzibur Rahman M. Recent development in metallic nanoparticles for breast cancer therapy and diagnosis. Chem Rec. 2022;22:e202100331. 35146897 10.1002/tcr.202100331

[23] Kim M, Lee JS, Kim W, Lee JH, Jun BH, Kim KS, et al. Aptamer-conjugated nano-liposome for immunogenic chemotherapy with reversal of immunosuppression. J Control Release. 2022;348:893–910. 35760233 10.1016/j.jconrel.2022.06.039

[24] Xu L, Cao Y, Xu Y, Li R, Xu X. Redox-responsive polymeric nanoparticle for nucleic acid delivery and cancer therapy: progress, opportunities, and challenges. Macromol Biosci. 2024;24:e2300238. 37573033 10.1002/mabi.202300238 PMC13420779

[25] Mi P. Stimuli-responsive nanocarriers for drug delivery, tumor imaging, therapy and theranostics. Theranostics. 2020;10:4557–88. 32292515 10.7150/thno.38069 PMC7150471

[26] Moreira AF, Dias DR, Costa EC, Correia IJ. Thermo- and pH-responsive nano-in-micro particles for combinatorial drug delivery to cancer cells. Eur J Pharm Sci. 2017;104:42–51. 28347775 10.1016/j.ejps.2017.03.033

[27] Yang Z, Zhang N, Ma T, Liu L, Zhao L, Xie H. Engineered bovine serum albumin-based nanoparticles with pH-sensitivity for doxorubicin delivery and controlled release. Drug Deliv. 2020;27:1156–64. 32755291 10.1080/10717544.2020.1797243 PMC7470134

[28] Meng X, Gui B, Yuan D, Zeller M, Wang C. Mechanized azobenzene-functionalized zirconium metal-organic framework for on-command cargo release. Sci Adv. 2016;2:e1600480. 27493996 10.1126/sciadv.1600480 PMC4972467

[29] Zhang J, Yang Y, Gao Y, Bai Z, Zhang X, Li K, et al. Non-interference delivery of Ce6 and DOX in NIR light-responsive liposomes for synergetic cervical cancer therapy. Biomed Mater. 2023;18. 10.1088/1748-605X/ace4b0. 10.1088/1748-605X/ace4b0 37406639

[30] Kadkhoda J, Tarighatnia A, Tohidkia MR, Nader ND, Aghanejad A. Photothermal therapy-mediated autophagy in breast cancer treatment: progress and trends. Life Sci. 2022;298:120499. 35346674 10.1016/j.lfs.2022.120499

[31] Winifred Nompumelelo Simelane N, Abrahamse H. Nanoparticle-Mediated Delivery Systems in Photodynamic Therapy of Colorectal Cancer. Int J Mol Sci. 2021;22:12405. 34830287 10.3390/ijms222212405 PMC8622021

[32] Xu W, Qian J, Hou G, Wang Y, Wang J, Sun T. A dual-targeted hyaluronic acid-gold nanorod platform with triple-stimuli responsiveness for photodynamic/photothermal therapy of breast cancer. Acta Biomater. 2019;83:400–13. 30465921 10.1016/j.actbio.2018.11.026

[33] Wang Y, Li M, Luo T, Jiao M, Jin S, Dou P, et al. Development of FL/MR dual-modal Au nanobipyramids for targeted cancer imaging and photothermal therapy. Mater Sci Eng C Mater Biol Appl. 2021;127:112190. 34225846 10.1016/j.msec.2021.112190

[34] Wen C, Guo X, Gao C, Zhu Z, Meng N, Shen XC, et al. NIR-II-responsive AuNRs@SiO(2)-RB@MnO(2) nanotheranostic for multimodal imaging-guided CDT/PTT synergistic cancer therapy. J Mater Chem B. 2022;10:4274–84. 35583909 10.1039/D1TB02807C

[35] Wang N, Zhao Z, Xiao X, Mo L, Yao W, Yang H, et al. ROS-responsive self-activatable photosensitizing agent for photodynamic-immunotherapy of cancer. Acta Biomater. 2023;164:511–21. 37004782 10.1016/j.actbio.2023.03.038

[36] Liu J, Cheng H, Han L, Qiang Z, Zhang X, Gao W, et al. Synergistic combination therapy of lung cancer using paclitaxel- and triptolide-coloaded lipid-polymer hybrid nanoparticles. Drug Des Dev Ther. 2018;12:3199–209. 10.2147/DDDT.S172199 PMC6161729 30288024

[37] Wen K, Wu L, Wu X, Lu Y, Duan T, Ma H, et al. Precisely tuning photothermal and photodynamic effects of polymeric nanoparticles by controlled copolymerization. Angew Chem Int Ed Engl. 2020;59:12756–61. 32343868 10.1002/anie.202004181

[38] Zhang YQ, Shen Y, Liao MM, Mao X, Mi GJ, You C, et al. Galactosylated chitosan triptolide nanoparticles for overcoming hepatocellular carcinoma: enhanced therapeutic efficacy, low toxicity, and validated network regulatory mechanisms. Nanomedicine. 2019;15:86–97. 30244085 10.1016/j.nano.2018.09.002

[39] Van Opdenbosch N, Lamkanfi M. Caspases in cell death, inflammation, and disease. Immunity. 2019;50:1352–64. 31216460 10.1016/j.immuni.2019.05.020 PMC6611727

[40] Martin SJ, Green DRGreen. protease activation during apoptosis: death by a thousand cuts? Cell. 1995;82:349–52. 7634323 10.1016/0092-8674(95)90422-0

[41] Doran AC, Yurdagul A Jr, Tabas I. Efferocytosis in health and disease. Nat Rev Immunol. 2020;20:254–67. 31822793 10.1038/s41577-019-0240-6 PMC7667664

[42] Abe T, Sakaguchi Y, Ohno S, Ikeda Y, Kitamura K, Maehara Y, et al. Apoptosis and p53 overexpression in human rectal cancer; relationship with response to hyperthermo-chemo-radiotherapy. Anticancer Res. 2001;21:2115–20. 11501834

[43] Suzuki K, Kazui T, Yoshida M, Uno T, Kobayashi T, Kimura T, et al. Drug-induced apoptosis and p53, BCL-2 and BAX expression in breast cancer tissues in vivo and in fibroblast cells in vitro. Jpn J Clin Oncol. 1999;29:323–31. 10470656 10.1093/jjco/29.7.323

[44] Smerage JB, Budd GT, Doyle GV, Brown M, Paoletti C, Muniz M, et al. Monitoring apoptosis and Bcl-2 on circulating tumor cells in patients with metastatic breast cancer. Mol Oncol. 2013;7:680–92. 23538216 10.1016/j.molonc.2013.02.013 PMC5528485

[45] Boyacıoğlu Ö, Bilgiç E, Varan C, Bilensoy E, Nemutlu E, Sevim D, et al. ACPA decreases non-small cell lung cancer line growth through Akt/PI3K and JNK pathways in vitro. Cell Death Dis. 2021;12:56. 33431819 10.1038/s41419-020-03274-3 PMC7801394

[46] Pessoa J. Cytochrome c in cancer therapy and prognosis. Biosci Rep. 2022;42:BSR20222171. 36479932 10.1042/BSR20222171 PMC9780037

[47] Battogtokh G, Cho YY, Lee JY, Lee HS, Kang HC. Mitochondrial-targeting anticancer agent conjugates and nanocarrier systems for cancer treatment. Front Pharm. 2018;9:922. 10.3389/fphar.2018.00922 PMC6107715 30174604

[48] Chernenko T, Matthäus C, Milane L, Quintero L, Amiji M, Diem M. Label-free Raman spectral imaging of intracellular delivery and degradation of polymeric nanoparticle systems. ACS Nano. 2009;3:3552–9. 19863088 10.1021/nn9010973

[49] Lakkadwala S, Singh J. Co-delivery of doxorubicin and erlotinib through liposomal nanoparticles for glioblastoma tumor regression using an in vitro brain tumor model. Colloids Surf B Biointerfaces. 2019;173:27–35. 30261346 10.1016/j.colsurfb.2018.09.047 PMC6296250

[50] George BP, Rajendran NK, Houreld NN, Abrahamse H. Rubus capped zinc oxide nanoparticles induce apoptosis in MCF-7 breast cancer cells. Molecules 2022;27:6862. 36296460 10.3390/molecules27206862 PMC9611499

[51] Hong EJ, Sivakumar P, Ravichandran V, Choi DG, Kim YS, Shim MS. Pro-oxidant drug-loaded Au/ZnO hybrid nanoparticles for cancer-specific chemo-photodynamic combination therapy. ACS Biomater Sci Eng. 2019;5:5209–17. 33455226 10.1021/acsbiomaterials.9b01339

[52] Levy JMM, Towers CG, Thorburn A. Targeting autophagy in cancer. Nat Rev Cancer. 2017;17:528–42. 28751651 10.1038/nrc.2017.53 PMC5975367

[53] Agarwal S, Maekawa T. Nano delivery of natural substances as prospective autophagy modulators in glioblastoma. Nanomedicine. 2020;29:102270. 32702467 10.1016/j.nano.2020.102270

[54] Yang X, Yang W, Xia X, Lei T, Yang Z, Jia W, et al. Intranasal delivery of BACE1 siRNA and rapamycin by dual targets modified nanoparticles for Alzheimer’s disease therapy. Small. 2022;18:e2203182. 35771092 10.1002/smll.202203182

[55] Hu L, Huang S, Chen G, Li B, Li T, Lin M, et al. Nanodrugs incorporating LDHA siRNA inhibit M2-like polarization of TAMs and amplify autophagy to assist oxaliplatin chemotherapy against colorectal cancer. ACS Appl Mater Interfaces. 2022;14:31625–33. 35796429 10.1021/acsami.2c05841

[56] Zhu M, Du L, Zhao R, Wang HY, Zhao Y, Nie G, et al. Cell-penetrating nanoparticles activate the inflammasome to enhance antibody production by targeting microtubule-associated protein 1-light chain 3 for degradation. ACS Nano. 2020;14:3703–17. 32057231 10.1021/acsnano.0c00962 PMC7457719

[57] Lu HY, Chang YJ, Fan NC, Wang LS, Lai NC, Yang CM, et al. Synergism through combination of chemotherapy and oxidative stress-induced autophagy in A549 lung cancer cells using redox-responsive nanohybrids: a new strategy for cancer therapy. Biomaterials. 2015;42:30–41. 25542791 10.1016/j.biomaterials.2014.11.029

[58] Deng Y, Song P, Chen X, Huang Y, Hong L, Jin Q, et al. 3-Bromopyruvate-conjugated nanoplatform-induced pro-death autophagy for enhanced photodynamic therapy against hypoxic tumor. ACS Nano. 2020;14:9711–27. 32806075 10.1021/acsnano.0c01350

[59] Lin L, Zhao Y, Zheng Q, Zhang J, Li H, Wu W. Epigenetic targeting of autophagy for cancer: DNA and RNA methylation. Front Oncol. 2023;13:1290330. 38148841 10.3389/fonc.2023.1290330 PMC10749975

[60] Newton K, Strasser A, Kayagaki N, Dixit VM. Cell death. Cell. 2024;187:235–56. 38242081 10.1016/j.cell.2023.11.044

[61] Zhao Q, Yang LJ, Zheng YB, Gong JH. Programmed necrosis inducers for cancer treatment. Zhongguo Yi Xue Ke Xue Yuan Xue Bao. 2022;44:338–47. 35538772 10.3881/j.issn.1000-503X.13241

[62] Newton K. RIPK1 and RIPK3: critical regulators of inflammation and cell death. Trends Cell Biol. 2015;25:347–53. 25662614 10.1016/j.tcb.2015.01.001

[63] Snyder AG, Hubbard NW, Messmer MN, Kofman SB, Hagan CE, Orozco SL, et al. Intratumoral activation of the necroptotic pathway components RIPK1 and RIPK3 potentiates antitumor immunity. Sci Immunol. 2019;4:eaaw2004. 31227597 10.1126/sciimmunol.aaw2004 PMC6831211

[64] Xu H, Zeng X, Wei X, Xue Z, Chen N, Zhu W, et al. Rosin derivative IDOAMP inhibits prostate cancer growth via activating RIPK1/RIPK3/MLKL signaling pathway. Oxid Med Cell Longev. 2022;2022:9325973. 35965682 10.1155/2022/9325973 PMC9371855

[65] Hänggi K, Vasilikos L, Valls AF, Yerbes R, Knop J, Spilgies LM, et al. RIPK1/RIPK3 promotes vascular permeability to allow tumor cell extravasation independent of its necroptotic function. Cell Death Dis. 2017;8:e2588. 28151480 10.1038/cddis.2017.20 PMC5386469

[66] Malumbres M. Cyclin-dependent kinases. Genome Biol. 2014;15:122. 25180339 10.1186/gb4184 PMC4097832

[67] Zhou Y, Lu L, Jiang G, Chen Z, Li J, An P, et al. Targeting CDK7 increases the stability of Snail to promote the dissemination of colorectal cancer. Cell Death Differ. 2019;26:1442–52. 30451989 10.1038/s41418-018-0222-4 PMC6748077

[68] Guan J, Guo H, Tang T, Wang Y, Wei Y, Seth P, et al. iRGD-liposomes enhance tumor delivery and therapeutic efficacy of antisense oligonucleotide drugs against primary prostate cancer and bone metastasis. Adv Funct Mater. 2021;31:2100478. 34211360 10.1002/adfm.202100478 PMC8240484

[69] Cai R, Wang M, Liu M, Zhu X, Feng L, Yu Z, et al. An iRGD-conjugated photothermal therapy-responsive gold nanoparticle system carrying siCDK7 induces necroptosis and immunotherapeutic responses in lung adenocarcinoma. Bioeng Transl Med. 2023;8:e10430. 37476070 10.1002/btm2.10430 PMC10354770

[70] Schwabe RF, Luedde T. Apoptosis and necroptosis in the liver: a matter of life and death. Nat Rev Gastroenterol Hepatol. 2018;15:738–52. 30250076 10.1038/s41575-018-0065-y PMC6490680

[71] Vucur M, Ghallab A, Schneider AT, Adili A, Cheng M, Castoldi M, et al. Sublethal necroptosis signaling promotes inflammation and liver cancer. Immunity. 2023;56:1578–1595.e8. 37329888 10.1016/j.immuni.2023.05.017

[72] Kroemer G, Galluzzi L, Kepp O, Zitvogel L. Immunogenic cell death in cancer therapy. Annu Rev Immunol. 2013;31:51–72. 23157435 10.1146/annurev-immunol-032712-100008

[73] Mathios D, Kim JE, Mangraviti A, Phallen J, Park CK, Jackson CM, et al. Anti-PD-1 antitumor immunity is enhanced by local and abrogated by systemic chemotherapy in GBM. Sci Transl Med. 2016;8:370ra180. 28003545 10.1126/scitranslmed.aag2942 PMC5724383

[74] Binnewies M, Roberts EW, Kersten K, Chan V, Fearon DF, Merad M, et al. Understanding the tumor immune microenvironment (TIME) for effective therapy. Nat Med. 2018;24:541–50. 29686425 10.1038/s41591-018-0014-x PMC5998822

[75] Guo W, Wu Z, Chen J, Guo S, You W, Wang S, et al. Nanoparticle delivery of miR-21-3p sensitizes melanoma to anti-PD-1 immunotherapy by promoting ferroptosis. J Immunother Cancer. 2022;10:e004381. 35738798 10.1136/jitc-2021-004381 PMC9226924

[76] Chen H, Luan X, Paholak HJ, Burnett JP, Stevers NO, Sansanaphongpricha K, et al. Depleting tumor-associated Tregs via nanoparticle-mediated hyperthermia to enhance anti-CTLA-4 immunotherapy. Nanomed (Lond.). 2020;15:77–92. 10.2217/nnm-2019-0190 PMC7132783 31868112

[77] Kranz LM, Diken M, Haas H, Kreiter S, Loquai C, Reuter KC, et al. Systemic RNA delivery to dendritic cells exploits antiviral defence for cancer immunotherapy. Nature. 2016;534(Jun):396–401. 27281205 10.1038/nature18300

[78] Krysko DV, Garg AD, Kaczmarek A, Krysko O, Agostinis P, Vandenabeele P. Immunogenic cell death and DAMPs in cancer therapy. Nat Rev Cancer. 2012;12:860–75. 23151605 10.1038/nrc3380

[79] Fucikova J, Spisek R, Kroemer G, Galluzzi L. Calreticulin and cancer. Cell Res. 2021;31:5–16. 32733014 10.1038/s41422-020-0383-9 PMC7853084

[80] Liu Y, Yan W, Tohme S, Chen M, Fu Y, Tian D, et al. Hypoxia induced HMGB1 and mitochondrial DNA interactions mediate tumor growth in hepatocellular carcinoma through Toll-like receptor 9. J Hepatol. 2015;63:114–21. 25681553 10.1016/j.jhep.2015.02.009 PMC4475488

[81] Jin Y, Zhuang Y, Dong X, Liu M. Development of CpG oligodeoxynucleotide TLR9 agonists in anti-cancer therapy. Expert Rev Anticancer Ther. 2021;21:841–51. 33831324 10.1080/14737140.2021.1915136

[82] Koster BD, López González M, van den Hout MF, Turksma AW, Sluijter BJ, Molenkamp BG, et al. T cell infiltration on local CpG-B delivery in early-stage melanoma is predominantly related to CLEC9A(+)CD141(+) cDC1 and CD14(+) antigen-presenting cell recruitment. J Immunother Cancer. 2021;9:e001962. 33737341 10.1136/jitc-2020-001962 PMC7978250

[83] Chen H, Zhang Y, Li L, Guo R, Shi X, Cao X. Effective CpG Delivery Using Zwitterion-Functionalized Dendrimer-Entrapped Gold Nanoparticles to Promote T Cell-Mediated Immunotherapy of Cancer Cells. Biosensors. 2022;12:71. 35200332 10.3390/bios12020071 PMC8869692

[84] Kleemann J, Steinhorst K, König V, Zöller N, Cinatl J Jr, Özistanbullu D, et al. Functional Downregulation of PD-L1 and PD-L2 by CpG and non-CpG oligonucleotides in melanoma cells. Cancers. 2022;14:4698. 36230620 10.3390/cancers14194698 PMC9562717

[85] Bertheloot D, Latz E, Franklin BS. Necroptosis, pyroptosis and apoptosis: an intricate game of cell death. Cell Mol Immunol. 2021;18:1106–21. 33785842 10.1038/s41423-020-00630-3 PMC8008022

[86] Nadeem S, Chen Z, Wei M, Li F, Ling D. Nanomedicine-induced pyroptosis for cancer therapy. Nanomedicine. 2021;16:1071–74. 33942673 10.2217/nnm-2021-0063

[87] Jin J, Yuan P, Yu W, Lin J, Xu A, Xu X, et al. Mitochondria-targeting polymer micelle of dichloroacetate induced pyroptosis to enhance osteosarcoma immunotherapy. ACS Nano. 2022;16:10327–40. 35737477 10.1021/acsnano.2c00192

[88] Wang H, He Z, Gao Y, Feng D, Wei X, Huang Y, et al. Dual-pronged attack: pH-driven membrane-anchored NIR dual-type nano-photosensitizer excites immunogenic pyroptosis and sequester immune checkpoint for enhanced prostate cancer photo-immunotherapy. Adv Sci. 2023;10:e2302422. 10.1002/advs.202302422 PMC10558672 37544896

[89] Li J, Ding B, Tan J, Chen H, Meng Q, Li X, et al. Sodium citrate nanoparticles induce dual-path pyroptosis for enhanced antitumor immunotherapy through synergistic ion overload and metabolic disturbance. Nano Lett. 2023;23:10034–43. 37903236 10.1021/acs.nanolett.3c03382

[90] Zhong H, Chen G, Li T, Huang J, Lin M, Li B, et al. Nanodrug augmenting antitumor immunity for enhanced TNBC therapy via pyroptosis and cGAS-STING activation. Nano Lett. 2023;23:5083–91. 37220198 10.1021/acs.nanolett.3c01008

[91] Liu Y, Lu Y, Ning B, Su X, Yang B, Dong H, et al. Intravenous delivery of living listeria monocytogenes elicits gasdmermin-dependent tumor pyroptosis and motivates anti-tumor immune response. ACS Nano. 2022;16:4102–15. 35262333 10.1021/acsnano.1c09818

[92] Wang Y, Liu T, Li X, Sheng H, Ma X, Hao L, et al. Ferroptosis-inducing nanomedicine for cancer therapy. Front Pharm. 2021;12:735965. 10.3389/fphar.2021.735965 PMC8722674 34987385

[93] Shen Z, Liu T, Li Y, Lau J, Yang Z, Fan W, et al. Fenton-reaction-acceleratable magnetic nanoparticles for ferroptosis therapy of orthotopic brain tumors. ACS Nano. 2018;12:11355–65. 30375848 10.1021/acsnano.8b06201

[94] Xiong H, Wang C, Wang Z, Lu H, Yao J. Self-assembled nano-activator constructed ferroptosis-immunotherapy through hijacking endogenous iron to intracellular positive feedback loop. J Control Release. 2021;332:539–52. 33689796 10.1016/j.jconrel.2021.03.007

[95] Nirmal GR, Lin ZC, Lin CH, Sung CT, Liao CC, Fang JY. Polydopamine/IR820 nanoparticles as topical phototheranostics for inhibiting psoriasiform lesions through dual photothermal and photodynamic treatments. Biomater Sci. 2022;10:6172–89. 36073349 10.1039/D2BM00835A

[96] Bai Z, Zhou Y, Peng Y, Ye X, Ma L. Perspectives and mechanisms for targeting mitotic catastrophe in cancer treatment. Biochim Biophys Acta Rev Cancer. 2023;1878:188965. 37625527 10.1016/j.bbcan.2023.188965

[97] Denisenko TV, Sorokina IV, Gogvadze V, Zhivotovsky B. Mitotic catastrophe and cancer drug resistance: a link that must to be broken. Drug Resist Updat. 2016;24:1–12. 26830311 10.1016/j.drup.2015.11.002

[98] Mu R, Wang YB, Wu M, Yang Y, Song W, Li T, et al. Depletion of pre-mRNA splicing factor Cdc5L inhibits mitotic progression and triggers mitotic catastrophe. Cell Death Dis. 2014;5:e1151. 24675469 10.1038/cddis.2014.117 PMC3973201

[99] Mlynarczuk-Bialy I, Dziuba I, Sarnecka A, Platos E, Kowalczyk M, Pels KK, et al. Entosis: from cell biology to clinical cancer pathology. Cancers. 2020;12:2481. 32883000 10.3390/cancers12092481 PMC7563411

[100] Liu J, Wang L, Zhang Y, Li S, Sun F, Wang G, et al. Induction of entosis in prostate cancer cells by nintedanib and its therapeutic implications. Oncol Lett. 2019;17:3151–62. 30867745 10.3892/ol.2019.9951 PMC6396220

[101] Zhao Z, Ma Z, Wang B, Guan Y, Su XD, Jiang Z. Mn(2+) directly activates cGAS and structural analysis suggests Mn(2+) induces a noncanonical catalytic synthesis of 2'3’-cGAMP. Cell Rep. 2020;32:108053. 32814054 10.1016/j.celrep.2020.108053

[102] Lazaro-Carrillo A, Calero M, Aires A, L Cortajarena A, Simões BM, et al. Tailored functionalized magnetic nanoparticles to target breast cancer cells including cancer stem-like cells. Cancers. 2020;12:1397. 32485849 10.3390/cancers12061397 PMC7352336

[103] Kunoh T, Shimura T, Kasai T, Matsumoto S, Mahmud H, Khayrani AC, et al. Use of DNA-generated gold nanoparticles to radiosensitize and eradicate radioresistant glioma stem cells. Nanotechnology. 2019;30:055101. 30499457 10.1088/1361-6528/aaedd5

[104] Zhang Y, Zhang L, Gao J, Wen L. Pro-death or pro-survival: contrasting paradigms on nanomaterial-induced autophagy and exploitations for cancer therapy. Acc Chem Res. 2019;52:3164–76. 31621285 10.1021/acs.accounts.9b00397

[105] Li H, Feng Y, Luo Q, Li Z, Li X, Gan H, et al. Stimuli-activatable nanomedicine meets cancer theranostics. Theranostics. 2023;13:5386–417. 37908735 10.7150/thno.87854 PMC10614691

[106] Linde MH, Fan AC, Köhnke T, Trotman-Grant AC, Gurev SF, Phan P, et al. Reprogramming cancer into antigen-presenting cells as a novel immunotherapy. Cancer Discov. 2023;13:1164–85. 36856575 10.1158/2159-8290.CD-21-0502

[107] Li W, Zhou C, Yu L, Hou Z, Liu H, Kong L, et al. Tumor-derived lactate promotes resistance to bevacizumab treatment by facilitating autophagy enhancer protein RUBCNL expression through histone H3 lysine 18 lactylation (H3K18la) in colorectal cancer. Autophagy. 2024;20:114–30. 37615625 10.1080/15548627.2023.2249762 PMC10761097

[108] Barbieri I, Kouzarides T. Role of RNA modifications in cancer. Nat Rev Cancer. 2020;20:303–22. 32300195 10.1038/s41568-020-0253-2

[109] Li X, Ma S, Deng Y, Yi P, Yu J. Targeting the RNA m(6)A modification for cancer immunotherapy. Mol Cancer. 2022;21:76. 35296338 10.1186/s12943-022-01558-0 PMC8924732

[110] Ngen EJ, Benham Azad B, Boinapally S, Lisok A, Brummet M, Jacob D, et al. MRI assessment of prostate-specific membrane antigen (PSMA) targeting by a PSMA-targeted magnetic nanoparticle: potential for image-guided therapy. Mol Pharm. 2019;16:2060–8. 30912947 10.1021/acs.molpharmaceut.9b00036

[111] Du D, Fu HJ, Ren WW, Li XL, Guo LH. PSA targeted dual-modality manganese oxide-mesoporous silica nanoparticles for prostate cancer imaging. Biomed Pharmacother. 2020;121:109614. 31731188 10.1016/j.biopha.2019.109614

[112] Jing H, Cheng W, Li S, Wu B, Leng X, Xu S, et al. Novel cell-penetrating peptide-loaded nanobubbles synergized with ultrasound irradiation enhance EGFR siRNA delivery for triple negative Breast cancer therapy. Colloids Surf B Biointerfaces. 2016;146:387–95. 27388967 10.1016/j.colsurfb.2016.06.037

[113] Pan Y, Volkmer JP, Mach KE, Rouse RV, Liu JJ, Sahoo D, et al. Endoscopic molecular imaging of human bladder cancer using a CD47 antibody. Sci Transl Med. 2014;6:260ra148. 25355698 10.1126/scitranslmed.3009457

[114] Luo Y, Li J, Hu Y, Gao F, Pak-Heng Leung G, Geng F, et al. Injectable thermo-responsive nano-hydrogel loading triptolide for the anti-breast cancer enhancement via localized treatment based on “two strikes” effects. Acta Pharm Sin B. 2020;10:2227–45. 33304788 10.1016/j.apsb.2020.05.011 PMC7715064

[115] Zheng S, Löw K, Wagner S, Yang X, von Briesen H, Zou S. Cytotoxicity of triptolide and triptolide loaded polymeric micelles in vitro. Toxicol Vitr. 2011;25:1557–67. 10.1016/j.tiv.2011.05.020 21640813

[116] Yu L, Wang Z, Mo Z, Zou B, Yang Y, Sun R, et al. Synergetic delivery of triptolide and Ce6 with light-activatable liposomes for efficient hepatocellular carcinoma therapy. Acta Pharm Sin B. 2021;11:2004–15. 34386334 10.1016/j.apsb.2021.02.001 PMC8343191

[117] Yu Z, Li X, Duan J, Yang XD. Targeted treatment of colon cancer with aptamer-guided albumin nanoparticles loaded with docetaxel. Int J Nanomed. 2020;15:6737–48. 10.2147/IJN.S267177 PMC7494387 32982230

[118] Zhao X, Liu X, Zhang P, Liu Y, Ran W, Cai Y, et al. Injectable peptide hydrogel as intraperitoneal triptolide depot for the treatment of orthotopic hepatocellular carcinoma. Acta Pharm Sin B. 2019;9:1050–60. 31649853 10.1016/j.apsb.2019.06.001 PMC6804453

[119] Liu L, Xiong X, Shen M, Ru D, Gao P, Zhang X, et al. Co-delivery of triptolide and curcumin for ovarian cancer targeting therapy via mPEG-DPPE/CaP nanoparticle. J Biomed Nanotechnol. 2018;14:1761–72. 30041722 10.1166/jbn.2018.2633

[120] Shi J, Ren Y, Ma J, Luo X, Li J, Wu Y, et al. Novel CD44-targeting and pH/redox-dual-stimuli-responsive core-shell nanoparticles loading triptolide combats breast cancer growth and lung metastasis. J Nanobiotechnol. 2021;19:188. 10.1186/s12951-021-00934-0 PMC8220850 34162396

[121] Zhang X, Chibli H, Mielke R, Nadeau J. Ultrasmall gold-doxorubicin conjugates rapidly kill apoptosis-resistant cancer cells. Bioconjug Chem. 2011;22:235–43. 21189001 10.1021/bc100374p

[122] Gibbens-Bandala B, Morales-Avila E, Ferro-Flores G, Santos-Cuevas C, Meléndez-Alafort L, Trujillo-Nolasco M. et al. (177)Lu-Bombesin-PLGA (paclitaxel): a targeted controlled-release nanomedicine for bimodal therapy of breast cancer. Mater Sci Eng C Mater Biol Appl. 2019;105:110043. 31546458 10.1016/j.msec.2019.110043

[123] Li Z, Deng Y, Sun H, Tan C, Li H, Mo F, et al. Redox modulation with a perfluorocarbon nanoparticle to reverse Treg-mediated immunosuppression and enhance anti-tumor immunity. J Control Release. 2023;358:579–90. 37172908 10.1016/j.jconrel.2023.05.013

[124] Cholujova D, Bujnakova Z, Dutkova E, Hideshima T, Groen RW, Mitsiades CS, et al. Realgar nanoparticles versus ATO arsenic compounds induce in vitro and in vivo activity against multiple myeloma. Br J Haematol. 2017;179:756–71. 29048129 10.1111/bjh.14974 PMC5705577

[125] Yadav N, Tripathi AK, Parveen A, Parveen S, Banerjee M. PLGA-quercetin nano-formulation inhibits cancer progression via mitochondrial dependent caspase-3,7 and independent FoxO1 activation with concomitant PI3K/AKT suppression. Pharmaceutics. 2022;14:1326. 35890222 10.3390/pharmaceutics14071326 PMC9323198

[126] Shen J, Dong J, Shao F, Zhao J, Gong L, Wang H, et al. Graphene oxide induces autophagy and apoptosis via the ROS-dependent AMPK/mTOR/ULK-1 pathway in colorectal cancer cells. Nanomedicine. 2022;17:591–605. 35394351 10.2217/nnm-2022-0030

[127] Chen Z, Ye X, Yuan K, Liu W, Liu K, Li Y, et al. Lycorine nanoparticles induce apoptosis through mitochondrial intrinsic pathway and inhibit migration and invasion in HepG2 cells. IEEE Trans Nanobiosci. 2022;21:549–59. 10.1109/TNB.2021.3132104 34851831

[128] Man S, Li M, Zhou J, Wang H, Zhang J, Ma L. Polyethyleneimine coated Fe(3)O(4) magnetic nanoparticles induce autophagy, NF-κB and TGF-β signaling pathway activation in HeLa cervical carcinoma cells via reactive oxygen species generation. Biomater Sci. 2020;8:201–11. 31664285 10.1039/C9BM01563A

[129] Taheriazam A, Abad GGY, Hajimazdarany S, Imani MH, Ziaolhagh S, Zandieh MA, et al. Graphene oxide nanoarchitectures in cancer biology: nano-modulators of autophagy and apoptosis. J Control Release. 2023;354:503–22. 36641122 10.1016/j.jconrel.2023.01.028

[130] Chen X, Yu Q, Liu Y, Sheng Q, Shi K, Wang Y, et al. Synergistic cytotoxicity and co-autophagy inhibition in pancreatic tumor cells and cancer-associated fibroblasts by dual functional peptide-modified liposomes. Acta Biomater. 2019;99:339–49. 31499197 10.1016/j.actbio.2019.09.003

[131] Wan HY, Chen JL, Zhu X, Liu L, Wang J, Zhu XM. Titania-coated gold nano-bipyramids for blocking autophagy flux and sensitizing cancer cells to proteasome inhibitor-induced death. Adv Sci. 2018;5:1700585. 10.1002/advs.201700585 PMC5867123 29593960

[132] Lin YX, Wang Y, Ding J, Jiang A, Wang J, Yu M, et al. Reactivation of the tumor suppressor PTEN by mRNA nanoparticles enhances antitumor immunity in preclinical models. Sci Transl Med. 2021;13:eaba9772. 34162754 10.1126/scitranslmed.aba9772 PMC8284983

[133] Khan MI, Mohammad A, Patil G, Naqvi SA, Chauhan LK, Ahmad I. Induction of ROS, mitochondrial damage and autophagy in lung epithelial cancer cells by iron oxide nanoparticles. Biomaterials. 2012;33:1477–88. 22098780 10.1016/j.biomaterials.2011.10.080

[134] Niu Y, Tang E, Zhang Q. Cytotoxic effect of silica nanoparticles against hepatocellular carcinoma cells through necroptosis induction. Toxicol Res. 2019;8:1042–9. 10.1039/c9tx00240e PMC7021192 32153770

[135] Sonkusre P, Cameotra SS. Biogenic selenium nanoparticles induce ROS-mediated necroptosis in PC-3 cancer cells through TNF activation. J Nanobiotechnol. 2017;15:43. 10.1186/s12951-017-0276-3 PMC5463494 28592284

[136] Mohammadalipour Z, Rahmati M, Khataee A, Moosavi MA. Differential effects of N-TiO(2) nanoparticle and its photo-activated form on autophagy and necroptosis in human melanoma A375 cells. J Cell Physiol. 2020;235:8246–59. 31989650 10.1002/jcp.29479

[137] Chen J, Zhang R, Tao C, Huang X, Chen Z, Li X, et al. CuS-NiS(2) nanomaterials for MRI guided phototherapy of gastric carcinoma via triggering mitochondria-mediated apoptosis and MLKL/CAPG-mediated necroptosis. Nanotoxicology. 2020;14:774–87. 32401088 10.1080/17435390.2020.1759727

[138] Huang Y, Hsu JC, Koo H, Cormode DP. Repurposing ferumoxytol: diagnostic and therapeutic applications of an FDA-approved nanoparticle. Theranostics. 2022;12:796–816. 34976214 10.7150/thno.67375 PMC8692919

[139] Kim KS, Choi B, Choi H, Ko MJ, Kim DH, Kim DH. Enhanced natural killer cell anti-tumor activity with nanoparticles mediated ferroptosis and potential therapeutic application in prostate cancer. J Nanobiotechnol. 2022;20:428. 10.1186/s12951-022-01635-y PMC9523925 36175895

[140] Zheng Y, Chen J, Song XR, Chang MQ, Feng W, Huang H, et al. Manganese-enriched photonic/catalytic nanomedicine augments synergistic anti-TNBC photothermal/nanocatalytic/immuno-therapy via activating cGAS-STING pathway. Biomaterials. 2023;293:121988. 36580716 10.1016/j.biomaterials.2022.121988

[141] Demaria O, De Gassart A, Coso S, Gestermann N, Di Domizio J, Flatz L, et al. STING activation of tumor endothelial cells initiates spontaneous and therapeutic antitumor immunity. Proc Natl Acad Sci USA. 2015;112:15408–13. 26607445 10.1073/pnas.1512832112 PMC4687570

[142] Guo W, Li Z, Huang H, Xu Z, Chen Z, Shen G, et al. VB12-Sericin-PBLG-IR780 nanomicelles for programming cell pyroptosis via photothermal (PTT)/photodynamic (PDT) effect-induced mitochondrial DNA (mitoDNA) oxidative damage. ACS Appl Mater Interfaces. 2022;14:17008–21. 35394753 10.1021/acsami.1c22804

[143] Ding B, Sheng J, Zheng P, Li C, Li D, Cheng Z, et al. Biodegradable upconversion nanoparticles induce pyroptosis for cancer immunotherapy. Nano Lett. 2021;21:8281–9. 34591494 10.1021/acs.nanolett.1c02790

